# Distinctive features of diffusion-controlled radiation defect recombination in stoichiometric magnesium aluminate spinel single crystals and transparent polycrystalline ceramics

**DOI:** 10.1038/s41598-020-64778-8

**Published:** 2020-05-08

**Authors:** A. Lushchik, E. Feldbach, E. A. Kotomin, I. Kudryavtseva, V. N. Kuzovkov, A. I. Popov, V. Seeman, E. Shablonin

**Affiliations:** 1 Institute of Physics, University of Tartu, W. Ostwald Str. 1, 50411 Tartu, Estonia; 2 Institute of Solid State Physics, University of Latvia, Kengaraga 8, Riga, LV-1063 Latvia

**Keywords:** Ceramics, Structure of solids and liquids

## Abstract

MgAl_2_O_4_ spinel is important optical material for harsh radiation environment and other important applications. The kinetics of thermal annealing of the basic electron (*F*, *F*^+^) and hole (*V*) centers in stoichiometric MgAl_2_O_4_ spinel irradiated by fast neutrons and protons is analyzed in terms of diffusion-controlled bimolecular reactions. Properties of MgAl_2_O_4_ single crystals and optical polycrystalline ceramics are compared. It is demonstrated that both transparent ceramics and single crystals, as well as different types of irradiation show qualitatively similar kinetics, but the effective migration energy *E*_a_ and pre-exponent *D*_0_ are strongly correlated. Such correlation is discussed in terms of the so-called Meyer-Neldel rule known in chemical kinetics of condensed matter. The results for the irradiated spinel are compared with those for sapphire, MgO and other radiation-resistant materials.

## Introduction

Spinel materials based on magnesium aluminate (MgAl_2_O_4_) possess unique physical and chemical properties which determines their widespread investigations and attracts increasing interest for their use in various scientific and industrial applications (see^1,2^ and references therein). In particular, MgAl_2_O_4_ single crystals and transparent polycrystalline ceramics (further called as optical ceramics) are planned to be exploited as diagnostics windows in future fusion devices^3,4^ or to be used as materials for different laser media^5^, phosphors for solid state lighting and 3D printing^6,7^, scintillators^8^, matrices for fiber-optic temperature sensors^9,10^, and even as a porous material for humidity sensors^11,12^. MgAl_2_O_4_ spinel materials are also used as a substrate for thin film growth^13,14^, alternative waste immobilization matrices^15,16^ or target materials in the nuclear transmutation of actinides^17^. For numerous nuclear applications, an extremely high tolerance against heavy irradiation or prolonged stay in harsh environment with practically no swelling^18,19^ is of particular importance.

Extremely high tolerance of MgAl_2_O_4_ against intense radiation at least partly results from the lattice structure. Stoichiometric magnesium aluminate spinel is an equimolar mixture of MgO and Al_2_O_3_. Oxygen ions form face-centered cubic structure, while Al^3+^ cations with relatively large ionic radius occupy a half of octahedral interstices and small-radius Mg^2+^ cations are located at every eighth tetrahedral site^20^. In addition to large empty space in such “normal” MgAl_2_O_4_, the cationic sublattice can be easily disordered due to the favorable conditions for the swapping of cation positions and the appearance of cations in “wrong” positions − Mg|_Al_ or Al|_Mg_. The formation energy of these so-called antisite defects (ADs), which are charged (-1 or +1) with respect to a regular lattice, is significantly lower than that for any other elementary lattice defects^21^. Just this circumstance explains the presence of a considerable concentration of ADs in as-grown single crystals and optical ceramics, while further irradiation even increases the degree of cation disorder, a kind of structural inversion.

The radiation effects in constituent parts of MgAl_2_O_4_ − MgO and Al_2_O_3_ binary oxides have been thoroughly studied (see^22–35^ and references therein) and the results on the microstructure, creation mechanisms and annealing kinetics of Frenkel defects (interstitial-vacancy pairs) and their simplest aggregates have been extended to magnesium aluminate single crystals and optical ceramics (see, e.g.^36–52^). Note that electron-type primary lattice defects, the *F*^+^ and *F* centers (one or two electrons in the field of an oxygen vacancy) clearly manifest themselves in optical spectra^36,38,43,49^, while the as-grown and radiation-induced trapped hole *V*-type centers have been reliably detected by the electron paramagnetic resonance (EPR) method^53–58^. Notably, the main experimental results belong to vacancy-containing structural defects, while complementary oxygen interstitials (not associated with some additional lattice defect/impurity) are still the most “hidden” defects in metal oxides and cation interstitials easily undergo transformation and, as a result, mainly exist in the form of ADs.

In radiation-resistant MgAl_2_O_4_, structural defects are mainly formed via the universal displacement mechanism connected with the elastic collisions of incident energetic particles with material atoms/ions. Nevertheless, this impact mechanism is solely responsible for radiation damage in metals and alloys induced by fast neutrons (i.e. neutral particles, see, e.g^59^. and references therein). The situation is more complicated in case of wide-gap material irradiation with charged particles, which spend a significant part of their energy on the formation of different electronic excitations. According to further studies^60–68^, especially favorable conditions for the creation of structural defects via ionization mechanisms (connected with different electronic excitations) occur within the tracks of ~GeV heavy ions. However, the defect creation mechanisms connected with the decay/recombination of anion excitons/relaxed electron-hole pairs, which ensure radiation damage in model alkali halide crystals (see, e.g.^69^), are not realized in the majority of metal oxides (including MgAl_2_O_4_), where the formation energy of a Frenkel defect pair exceeds the energy gap, *E*_FD_ > *E*_g_. Therefore, less stable and more energetic electronic excitations (e.g., hot, non-relaxed e-h pairs^63,64,66–68^) could contribute to radiation damage under metal oxide irradiation with swift ions (including protons).

The present paper is devoted to the measuring of the thermal annealing kinetics of structural defects, both electron (*F*^+^ and *F* centers) and hole origin (paramagnetic *V* centers), induced in MgAl_2_O_4_ single crystal and optical ceramics by fast fission neutrons or 100-keV protons with varying fluence. The annealing curves are also simulated in terms of diffusion-controlled bimolecular reactions, and the features of the obtained kinetic parameters are considered and compared with those for other wide-gap materials. It is notable that kinetic parameters provide indirect information just on “poorly studied” oxygen and cation interstitials, which are acting as mobile components in the recombination process with vacancy-containing complementary Frenkel defects.

Note that the present study is a logical continuation of our previous investigations regarding the experimental analysis of electronic excitations and structural defects in magnesium aluminate spinel with different stoichiometry^57,58,70,71^ as well as a recent phenomenological theory of diffusion-controlled recombination of Frenkel defects in wide-gap materials^72–78^.

## Methods used

### Experimental

Nominally pure MgAl_2_O_4_ single crystals (i.e. a stoichiometric spinel) were grown by Union Carbide Corporation using the Czochralski method and irradiated by fast fission neutrons with fluence of *Φ* = 10^17^ *n/*cm^2^ and *Φ* = 2.6  ×  10^18^ *n*/cm^2^ at Oak Ridge National Laboratory (neutron energy > 1 MeV and irradiation temperature *T* ≤ 60 °C). Single crystals contained several dozen ppm of iron ions as well as the traces of Cr, Ca, and Mn impurities (see also^38^).

Transparent polycrystalline ceramics of MgAl_2_O_4_ with a grain size of 0.5, 1.4 and 12 μm were sintered by hot isostatic pressing in argon at 1510−1600 °C (lower value for the smallest grain size, upper − for 12-μm grains) at the Fraunhofer Institute for Ceramic Technologies and Systems. The lowest grain size was achieved by addition of a special doping, while a coarser spinel raw powder was used for the highest grain size. Stoichiometric ceramic samples contained impurities below 100 ppm in total, had inversion parameter about 0.4 (estimated on the basis of the lattice parameter determined by XRD^78^) and were in the form of polished 1-mm thick plates. The similar parameter of cation disorder in single crystals was lower, about 0.1–0.2.

The MgAl_2_O_4_ optical ceramics were irradiated with the 100-keV protons and *Φ* = (1-5) × 10^17^ *p*/cm^2^ using KIIA 500 kV ion implanter of Accelerator Laboratory at the University of Helsinki (see also^71^). According to SRIM simulation^79^, the penetration depth of such protons equals about 0.5 μm, while the radiation damage level exceeds ~1 dpa.

The spectra of optical absorption in a spectral region from 1.5 to 6.5 eV were measured by a spectrometer JASCO V-660, while a homemade setup equipped with a vacuum monochromator VMR-2 and the hydrogen discharge source allowed performing measurements up to 10 eV. The EPR measurements were made with an X-band (9.8 GHz) spectrometer Bruker ELEXYS-II E500. This study of paramagnetic centers was performed for a neutron-irradiated (*Φ* = 2.7  ×  10^18^ *n*/cm^2^) single crystal in the form of rectangular parallelepiped, one [110] axis of which was parallel to the magnetic field (see^57^ for details).

The stepwise thermal annealing of radiation damage was performed in the following way: *(i)* a porcelain boat with the irradiated samples was placed inside a quartz reactor of a furnace; *(ii)* heated from room temperature (RT) to a certain temperature *T*_pr_ in an argon atmosphere; *(iii)* kept at this *T*_pr_ for 10 min; and then *(iv)* cooled down by moving the reactor out of the furnace. Multiple “heating-cooling-measuring” cycles were executed under the same conditions with the rise of *T*_pr_ by 20–40 K for all samples preliminary irradiated with protons or fast neutrons. Note that all optical absorption and EPR spectra for the samples subjected to the ascribed annealing procedure were measured at RT.

### Theoretical

In order to analyze the experimental recombination kinetics, we have recently developed^72–76^ a simple phenomenological theory that takes into account the mutual diffusion-controlled approach to the electron and hole centers and their recombination. This model allows us to extract the key diffusion parameters – the effective migration energy *E*_a_ of more mobile species and the pre-exponential factor *X* = *N*_0_*RD*_0_/*β*, where *N*_0_ is initial defect concentration, *R* recombination radius, *D*_0_ pre-exponent for diffusion, and *β* heating rate upon defect thermal annealing. The typical value of the pre-exponential parameter for a normal solid state diffusion^72,74^ is *X* = 10^1^° K^−1^ if *N*_0_ = 10^19^ cm^−3^. This theory was successfully applied to sapphire, MgO and MgF_2_ crystals irradiated by energetic electrons, neutrons, protons or heavy ions^72,74–77^ and is used in the present study for MgAl_2_O_4_ spinel.

It is also well known that the *F* center in МgО and Al_2_O_3_ is immobile until ca. 1500 K^22^, so just its complementary defect – an interstitial oxygen ion – is significantly more mobile and reaches the *F*-type center before mutual recombination^72,74^. The situation in MgAl_2_O_4_ spinel is more unclear. We will try to discuss the contradictory results in this work.

## Results

Irradiation of MgAl_2_O_4_ crystals by high-energy particles and quanta causes the creation of different color centers/radiation defects responsible for the appearance of several optical absorption bands within the transparency region of virgin (non-irradiated) samples. As a result, the functionality of optical materials/components strongly depends on the radiation damage kinetics − accumulation of radiation defects with the irradiation fluence/dose as well as their annealing (i.e., recovery of optical properties) via a subsequent thermal treatment of the irradiated materials. In the present study, we analyzed the changes in the spectra of optical absorption caused by both the irradiation of MgAl_2_O_4_ single crystals/optical ceramics with fast neutrons or 100-keV protons and the subsequent thermal annealing of radiation damage in the irradiated samples.

**Figure 1:**
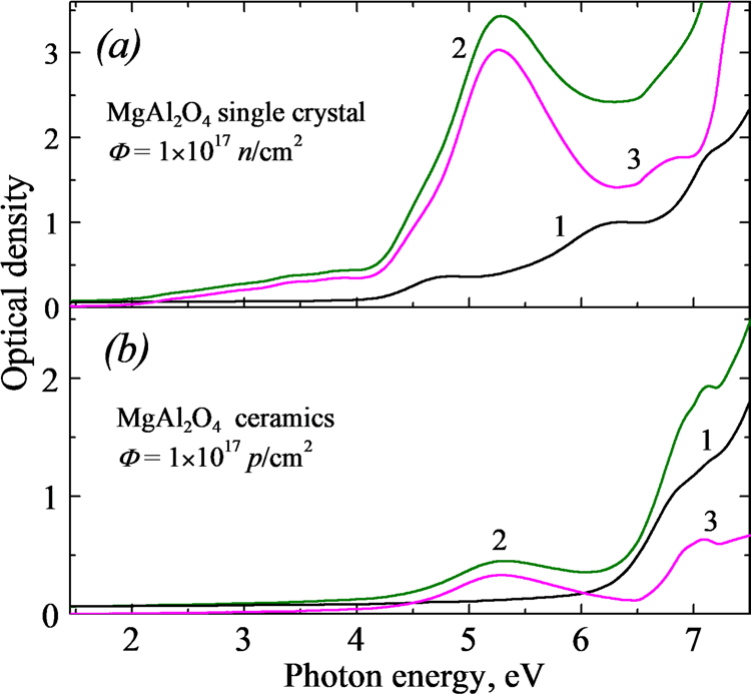
The absorption spectra of an MgAl_2_O_4_ single crystal (**a**) or ceramic sample (**b**), grain size of 12 μm) measured at 295 K before (curves 1) and after irradiation with fast neutrons or 100-keV protons (curves 2). The difference spectra (curves 3) represent radiation-induced optical absorption (RIOA).

Figure 1 presents, as an example, the spectra of optical absorption measured in a wide spectral region of 1.5–7.5 eV for a MgAl_2_O_4_ sample before (curve 1) and after irradiation (curve 2). The difference between these two absorption spectra has been considered as the radiation-induced optical absorption (RIOA, curve 3) representing the number (concentration) of the corresponding radiation defects. Figure 1a shows the spectra for a single crystal with thickness of *d* = 0.86 mm exposed to fast fission neutrons (*Φ* = 10^17^ *n/*cm^2^), while Fig. 1b is related to the irradiation of a transparent ceramic sample with 100-kev protons (*Φ* = 10^17^ *p/*cm^2^). Note that rather different values of RIOA around 5 eV in parts *(a)* and *(b)* reflect the homogeneous coloration through the whole sample thickness in case of neutron irradiation and a rather small amount of *F*-type centers formed in a thin sample layer (~ 0.5 μm) by 100-keV protons.

According to the literature^36–38,43,49,53^, a broad complex RIOA band peaked around 5.3 eV is mainly connected with electron-type *F*^+^ and *F* centers and the hole-containing color centers are responsible for the RIOA at ~3–4 eV. Note that the existing impurities, mostly Fe, Cr, Mn contribute to the absorption spectra as well (from IR to UV spectral region, see^36–38,50,51^ and references therein). Of particular interest is the origin of optical absorption above 6.5 eV, near the tentative edge of fundamental absorption. Despite the long-term investigations of MgAl_2_O_4_, even such fundamental characteristics of an insulating material as the value of the energy gap *E*_g_ was not precisely determined. According to the recent studies of MgAl_2_O_4_ single crystals and optical ceramics by means of several optical methods^70,71^, the *E*_g_ value at low temperature slightly exceeds 8 eV. In addition, the analysis of the excitation spectra for different emissions and phosphorescence allowed us to conclude that the spectral region of 6.8–7.4 eV is typical of the formation of excitonic-like states, i.e. such exciting photons cause the formation of bound excitons near charged ADs^70,71^. This suggestion is supported by the peak of RIOA at about 7 eV revealed during the process of thermal annealing of neutron- or proton-irradiated MgAl_2_O_4_ samples (see^57,71^).

**Figure 2:**
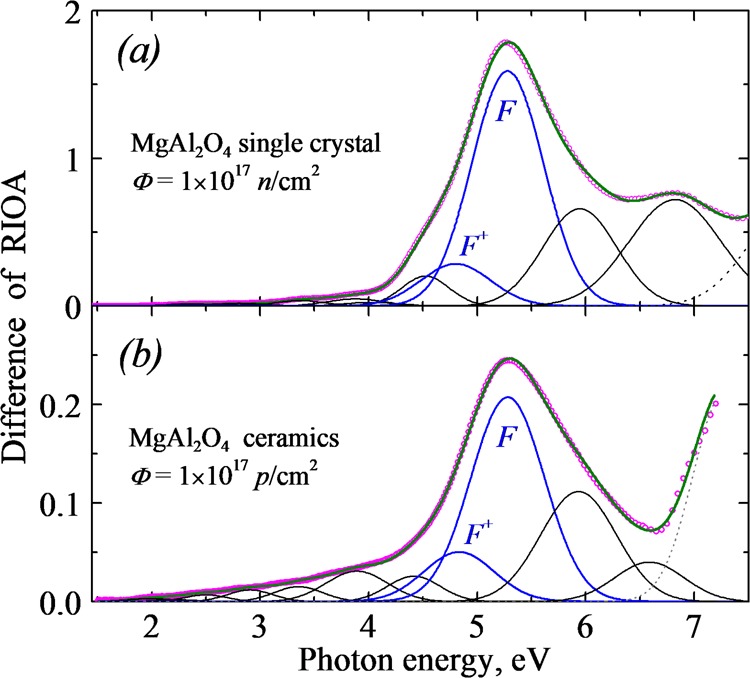
The decomposition of RIOA difference spectra into Gaussian components (thin lines) for an MgAl_2_O_4_ single crystal (**a**) or ceramic sample (**b**, grain size of 12 μm)) irradiated by fast neutrons and 100-keV protons, respectively. The difference spectra represent the decrease of RIOA caused by the preheated of the irradiated sample from 490 to 697 K (part *a*) and from 546 to 747 K (*b*). Symbols (ooo) depict experimental points and solid line − a fitted curve.

Figure 2 demonstrates the complexity of the main RIOA band peaked around 5.25 eV. Figure 2a depicts a difference spectrum that reflects the decrease of RIOA caused by fast-neutron-irradiation as a consequence of a single crystal preheating from 490 to 697 K (see curve with symbols − ooo). This spectrum is decomposed into several elementary Gaussian components, the parameters of which (integrated area *S* or peak intensity *I*_max_) can be considered to be a measure of the number of corresponding defects. As it has been already stated, we are interested in the annealing kinetics of electron-type *F*^+^ and *F* centers with well-defined characteristics of optical absorption. Therefore, other Gaussian components, a part of which is related to ADs or as-grown impurities and other structural defects, are not a subject of the present paper.

In case of predominance of a certain radiation defect (according to Fig. 2 − *F* centers are such radiation-induced defects in our case), there is a very small difference in thermal annealing curves for the *F* centers, the number of which has been estimated in three different ways: via *I*_max_ (in units of optical density) and *S* or even as the value of optical density (without decomposition into Gaussians) measured at photon energy where the contribution of a certain defect to RIOA is dominant. This conclusion, important from methodological point of view, has been proved recently for the case of *F*-type center annealing in MgO single crystals irradiated with swift heavy ions^80^. The accuracy of the estimation of the number of *F*^+^ centers in MgAl_2_O_4_ is certainly lower. Nevertheless, it is possible to select a certain *hν* where just the *F*^+^ centers are mainly responsible for optical absorption.

### The electronic centers

The results of theoretical analysis of annealing kinetics for the *F* and *F*^+^ centers created by fast neutrons or protons are summarized in Table 1. As one can see, the measurements were performed for both single crystals and optical ceramics with different grain size; moreover, different radiation fluences were used. The annealing kinetics was analyzed for both the electron (*F*^+^, *F*) and hole (*V*_1_, *V*_2_) centers.

**Table 1:** Explanation of curves I-V in Fig. 3 and the values of calculated migration energy *E*_a_ and pre-exponential factor *X* obtained under different irradiation conditions for the electron (Nos.1–12) and hole (Nos. 13 and 14) centers.

No.	Irradiation	Defect	*E*_a_ (eV)	*X* (K^−1^)	Legend
1 (I)	neutron	*F*	0.38	1.0 × 10^1^	Optical absorption, single crystal, 1 MeV, *Φ* = 1.0 × 10^17^ *n*/cm^2^
2	neutron	*F*^+^	0.35	5.1 × × 10^0^	same as No. 1
3 (II)	neutron	*F*	0.44	1.3 × 10^1^	Optical absorption, single crystal, 1 MeV, *Φ* = 2.6 × 10^18^ *n*/cm^2^,
4	neutron	*F*^+^	0.35	3.0 × 10^0^	same as No. 3
5 (III)	protons	*F*	0.60	1.4 × 10^2^	Optical absorption, ceramics with grain size 12 μm, 100 keV, *Φ* = 1.0 × 10^17^ *p*/cm^2^
6	protons	*F*^+^	0.58	9.4 × 10^1^	same as No. 5
7 (IV)	proton	*F*	0.24	8.5 × 10^−2^	Optical absorption, ceramics with grain size 1.4 μm, 100 keV, *Φ* = 1.0 × 10^17^ *p*/cm^2^
8	proton	*F*^+^	0.24	8.7 × 10^−2^	same as No. 7
9	proton	*F*	0.29	3.4 × 10^−1^	Optical absorption, ceramics with grain size 1.4 μm, 100 keV, *Φ* = 5.0 × 10^17^ *p*/cm^2^
10	proton	*F*^+^	0.22	9.3 × 10^−2^	same as No 9
11	proton	*F*	0.34	1.1 × 10^0^	Optical absorption, ceramics, grain size 0.5 μm, 100 keV, *Φ* = 2.0 × 10^17^ *p*/cm^2^
12	proton	*F*^+^	0.38	3.1 × 10^0^	same as No. 11
13 (V)	neutron	*V*_2_	0.64	2.5 × 10^2^	EPR signal, single crystal 1 MeV, *Φ* = 2.6 × 10^18^ *n*/cm^2^
14	neutron	*V*_1_	0.63	1.9 × 10^5^	same as No. 13

**Figure 3:**
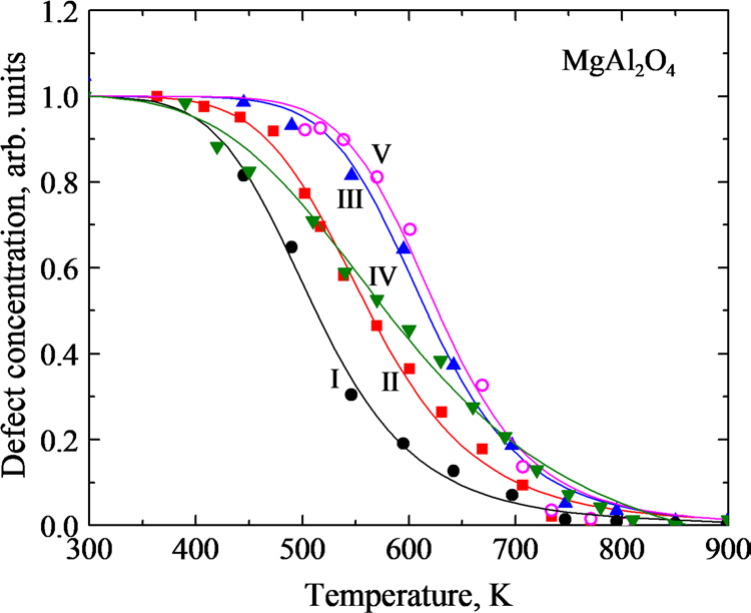
Kinetics of the *F* and *V*_2_ center annealing in MgAl_2_O_4_ samples (see Table 1 for details, only five representative kinetics are plotted here for illustration).

Typical annealing kinetics for the *F* centers after neutron and proton irradiation are shown in Fig. 3 (curves I-II and III-IV, respectively). These kinetics reveal monotonous and smooth decay of defect number (concentration) with temperature, similar to the previous study for sapphire, MgO and MgF_2_^74^. Note that the decay kinetics of the *F*^+^ centers in spinel samples is very similar to that for *F* centers and is not presented in Fig. 3. Because of rather large grain size of the used optical ceramics. In addition, there is no noticeable difference in the decay kinetics of *F*-type centers in our optical ceramics with different but rather large grain size. It is not surprising, because a “grain-size effect” (if any) could be expected in a real nanometer scale region (definitely below 100 nm). Theoretical curves (solid lines) fit very well to the experimental points. This allows us to extract by means of square fitting two key migration parameters, *E*_a_ and *X* with high accuracy.

**Figure 4:**
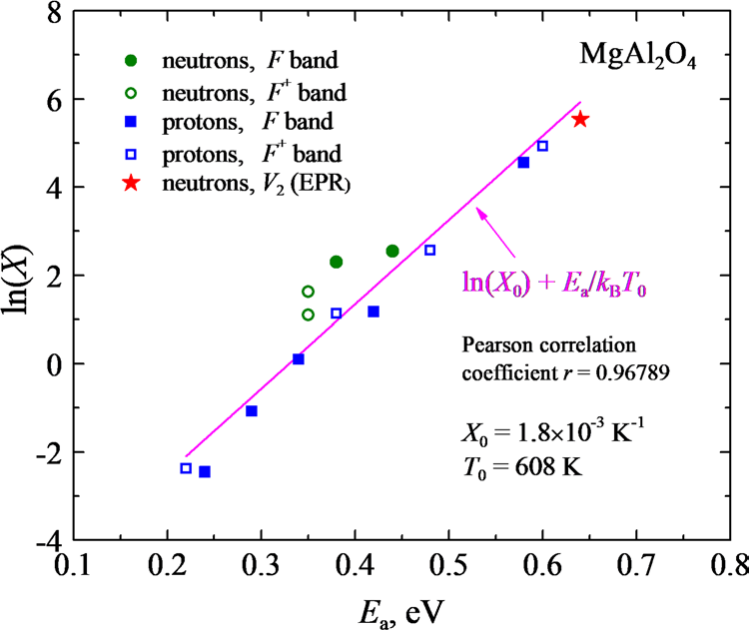
Correlation of the effective migration energies *E*_a_ and pre-exponents *X* for radiation defect annealing in MgAl_2_O_4_. The high quality of this correlation is characterized by the standard Pearson correlation coeffcient, which is very close to the perfect correlation case (*r* = 1).

As one can see in Table 1, both parameters reveal considerable variations, dependent on experimental conditions. The most surprising thing is that the parameter *X* for all experimental conditions is much smaller than that in the previously mentioned estimate for a normal solid-state diffusion, *X* = 10^1^° K^−1^ (*N*_0_ = 10^19^ cm^−3^)^72,74^. Moreover, these two parameters, *E*_*a*_ and *X*, show a strong correlation when plotted in the coordinates *ln(X)* versus *E*_*a*_ (see Fig. 4): the smaller energy, the smaller the relevant pre-exponential. It is noteworthy that bimolecular recombination rate contains a product *X·exp(−E*_a_*/k*_B_*T)*, this is why *ln(X)* instead of *X* is correlated with *E*_a_. The observed linear correlation is fulfilled very well within eight orders of magnitude in *X*; the standard Pearson correlation coefficient is almost one. The similar strong correlation of the migration energy and pre-exponential factor we have recently observed in several other radiation-resistant ionic solids – Al_2_O_3_, MgO, MgF_2_^74,77^. This type of correlation is known in chemical kinetics as *Mayer-Neldel rule* (MNR)^81–83^

1 $$ln(X)=ln({X}_{0})+{E}_{a}/{k}_{B}{T}_{0},$$

where *X*_0_ is constant and *T*_0_ characteristic temperature. In other words, the diffusion pre-exponent *X* turns out to be exponentially dependent on the migration energy. As a result, the effective diffusion coefficient now reads:

2 $$D \sim exp({E}_{a}/{k}_{B}[1/{T}_{0}-1/T]).$$

Despite confirmation of the MNR in this paper, there is considerable difference between spinel samples analyzed here and three above-mentioned ionic solids^74^. For instance, the obtained interstitial migration energy of 0.89 eV obtained at low fluences in α-Al_2_O_3_ is close to the calculated *E*_a_ for charged oxygen interstitials in a regular crystal, 0.8–1.0 eV^84^ (for neutral interstitial atoms the calculated energy barrier is higher, 1.3 eV). Similarly, the migration energy of 1.74 eV obtained in MgO from the experiment at low fluences is again close to the calculated 1.5 eV^85^. Lastly, for the lowest radiation dose of MgF_2_, the migration energy is also quite high, 1.74 eV. Moreover, high migration energies are accompanied by large pre-exponetials *X*: 1.4 × 10^5^ K^−1^ (for MgO) or 4.8 × 10^10^ K^−1^ (for MgF_2_) comparable to the above-mentioned estimate for a perfect solid, *X*~10^10^ K^−1^. These kinetic parameters corresponding to the low fluences and almost perfect solids are located in the upper right corner of the MNR diagram (see Fig. 4). With the fluence increase, the relevant energies and pre-exponentials move along the line to the low-left corner of the diagram.

However, defects in MgAl_2_O_4_ show quite different pattern: for all possible fluences both migration energy and pre-exponential are small and far from those expected for a crystalline solid, e.g. *E*_a_ ~ 1.0 eV calculated for interstitial oxygen ions in spinel^52^. This circumstance indicates to some new defect annealing mechanism. Note that the MNR is typically observed in strongly disordered systems^81–83^. In amorphous solids and liquids^86,87^, the particle (defect) diffusion jumps over energy barriers are replaced by a collective motion of atoms surrounding the defect; thermal density fluctuations create cavities for particle migration with low energy and low pre-exponential factor (rare events). To some degree, strongly irradiated solids could be considered as partly disordered, which results in MNR observation.

### Hole centers

Besides RIOA at 3–4 eV ascribed in the literature to *V* centers^36,37,43^, several hole-containing paramagnetic defects complementary to the *F*-type electron centers have been revealed in neutron-irradiated (*Φ* = 2.6 × 10^18^ *n/*cm^2^) MgAl_2_O_4_ single crystals by means of the EPR method (see also^57,58^). On the basis of *g*-factor value, angular dependence of the measured EPR spectra and the modelling program EasySpin^88^ it was proved that all revealed paramagnetic centers are of hole-type, while a hole is trapped at a regular oxygen ion O^2-^ (forming O^-^ ion) next to some negatively charged defect. In case of so-called *V*_1_ and *V*_2_ centers, such negative defect, formed under neutron irradiation, appeared to be an aluminium and magnesium vacancy, respectively (V_Al_ and V_Mg_).

The pulse annealing of the EPR signal of these centers with the model of *V*_1_ ≡ O^–^V_Al_ and *V*_2_ ≡ O^–^V_Mg_ was performed under the same conditions as the described above thermal annealing of RIOA. It was stated in^57,58^ that *V*_1_ and *V*_2_ radiation-induced defects decay irreversibly (as a kind of ionic process) and these structural defects cannot be restored by the subsequent X-ray irradiation (i.e. via recharging of potentially available as-grown cation vacancies by X-ray-induced charge carriers).

Curve V in Fig. 3 presents theoretical fitting (solid line) of the measured (experimental points are given by symbols) pulse annealing curve for the EPR signal of the *V*_2_ defects in a neutron-irradiated MgAl_2_O_4_ single crystal, whereas Table 1 gives the *E*_a_ and *X* for both hole centers, *V*_1_ and *V*_2_. According to Table 1 (Nos. 13 and 14), the values of *E*_a_ are very close for both hole centers (0.64 eV) but differ from those for *F*-type centers in the same neutron-irradiated MgAl_2_O_4_ crystals (0.44 eV). On the other hand, pre-exponential factors *X* for these hole centers vary by three orders of magnitude staying again in all cases significantly smaller than a typical value of *X* = 10^10^ K^−1^ ^72^. The difference in *E*_a_ values is reasonable because, in contrast to the annealing of *F*-type centers (see the previous Section), cation interstitials play the role of a mobile recombination component: becoming mobile they reach and fulfil complementary cation vacancies which are constitutive of neutron-induced *V*_1_ and *V*_2_ centers. It is important to remind that, by energetic reasons, cation interstitials from neutron-induced Frenkel pairs in spinel crystals undergo transformation into antisite defects with the lowest formation energy^21^. Note that the *V*_2_ center decay parameters fit well to the MNR (Fig. 4), which indicates to a strong correlation between the electron and hole center annealing.

### Concluding remarks

This study shows that the annealing of the electron centers in spinel, unlike parent binary oxides (MgO, Al_2_O_3_), is a multistage process. Based on the EPR-proved microstructure of paramagnetic defects and their thermal stability, we can conclude that, upon heating of the irradiated sample to ~400 K, Al^3+^ interstitials from Al|_Mg_ are the first defects to become mobile and able to fill-in an aluminum vacancy of the *V*_1_ defect producing irreversible decay of these radiation defects. At temperatures above ~500 K, Mg^2+^ interstitials from Mg|_Al_ antisite defects in their turn become mobile, reach *V*_2_ centers and destroy them via fulfilling magnesium vacancies. Probably, the observed energies (Nos. 13 and 14 in Table 1) correspond to these processes.

Note that both decay processes of *V*_1_ and *V*_2_ defects are accompanied by the decrease of the number of *F*-type centers (continues attenuation of the corresponding RIOA at *T* = 380–850 K). After filling the cation vacancies, the holes are released from *V*_1_ and *V*_2_ centers and could recombine with the electrons from the *F* and *F*^+^ centers. However, the irreversible annealing of RIOA responsible for the *F* and *F*^+^ centers occurs simultaneously and without any sign of recharging of these centers. Note that identical thermal annealing of *F*^+^ and *F* centers takes place in MgO crystals irradiated by swift heavy ions as well^80^. Therefore, the final destruction of the *F* and *F*^+^ centers or their products requires participation of oxygen interstitials which could be spatially correlated to the *F* and *F*^+^ centers. Alternatively, the mobile holes could release the trapped oxygen interstitials (e.g., at antisite defects) and stimulate their recombination with the *F*-type centers. However, this would need the energies of the order of 1 eV, much higher than those observed here. Thus, the physical meaning of the energies for the *F*-type centers in Table 1 remain unclear yet.

Note that the elastic collisions of fast neutrons with crystal atom nuclei lead to the creation of interstitial-vacancy Frenkel pairs in anion and cation sublattice. The involvement of both oxygen and cation interstitials is needed to describe the tentative scenario of the annealing of paramagnetic hole-containing centers in MgAl_2_O_4_ single crystal. A search for experimental manifestations of oxygen interstitials in MgAl_2_O_4_ (as well as in other binary and complex metal oxides) and the elucidation of their microstructure, preferable charge states, diffusion and trapping mechanisms still lies ahead.
